# From the year of the coronavirus disease 2019 (COVID-19) pandemic, the rise of *Antimicrobial Stewardship and Healthcare Epidemiology* (ASHE): Reflections and future priorities

**DOI:** 10.1017/ash.2021.168

**Published:** 2021-06-23

**Authors:** Gonzalo Bearman

**Affiliations:** Division of Infectious Diseases, Virginia Commonwealth University Health, Richmond, Virginia

“*I believe that business is nothing more than a group of people trying to make a difference*.”

—Sir Richard Branson

The launch of the open-access *Antimicrobial Stewardship and Healthcare Epidemiology* (ASHE) journal is a momentous step for the scientific mission of the Society for Healthcare Epidemiology of America. All of us on the editorial team are both honored and deeply excited about the future of ASHE. Before looking forward, it is important for us to pause and reflect on the multiple events of the last year and the coronavirus disease 2019 (COVID-19) pandemic to gain a better perspective on and clear commitment to what lies ahead.

The COVID-19 pandemic affected us in multiple ways. As healthcare epidemiologists and healthcare providers across varied components of the healthcare delivery spectrum, we faced tremendous challenges in infection prevention, patient quality and safety antimicrobial stewardship, disinfection, the use of personal protective equipment, diagnostic testing and the clinical management of SARS-CoV-2 infected patients. All of this was coupled with rapid scientific discovery and dissemination in the medical literature.

The COVID-19 pandemic further exposed the many weaknesses in the fabric of society, including significant healthcare disparities by race and inequalities in access and outcomes for individuals with COVID-19. The tragic death of George Floyd reignited an intense awareness of racism, diversity, justice, equity, and intersectionality. The year 2020 also witnessed an explosion of misinformation (ie, infodemic) across multiple spheres including medicine and politics. More than ever, the medical infodemic underscored the important role of epidemiologists as both content experts and scientists to drive an evidence-based public health response in critical times.

These unique times shaped the vision of ASHE. The journal is committed to the rapid dissemination of high-quality, timely, evolving science in antimicrobial stewardship and healthcare epidemiology in an open-access model, to serve both developed and low- and middle-income countries. To do so, we are committed to diversity in professional backgrounds, nationalities, and gender across the editorial team, such that journal leadership reflects not only the membership of the Society for Healthcare Epidemiology of America but also the antimicrobial stewardship and healthcare epidemiology community at large. Next, we will seek innovative original science, reviews, and commentaries that represent a diversity of perspectives, issues, evolving challenges, and controversies in the varied field of healthcare epidemiology and antimicrobial stewardship, with the ultimate goal of maximal content accessibility to a worldwide readership.

Authors submitting to ASHE will find that the journal provides constructive peer review, competitive turnaround times, immediate online publication, and social media promotion. As a fully gold open-access journal, ASHE readers will benefit from free and easy access to all journal content, bringing the widest possible impact, reach, and discoverability for our authors and readers. The journal is engaged in efforts to ensure that article processing charges (APCs) are not a barrier to publication. Our publisher, Cambridge University Press, has recently signed a number of read-and-publish agreements with hundreds of institutions worldwide, which will cover APCs for corresponding authors from those institutions. APCs for authors from low- or middle-income countries will be waived or discounted through initiatives like Research4Life^1^ Additionally, SHEA members will benefit from a highly discounted rate and the journal will waive publishing costs for invited reviews.

As we move forward and view the COVID-19 pandemic in the rearview mirror, we must not forget the valuable lessons learned from scientific responsiveness and the publication of high-quality science, under a vigorous peer review, in an open-access manner. With a diversity of both content and contributors, ASHE will aim for a global audience for advancing patient safety, antimicrobial stewardship, and healthcare epidemiology, with the aspiration of making a difference.
